# Ultrafast core-to-core luminescence in BaF_2_ - LaF_3_ single crystals

**DOI:** 10.1038/s41598-025-11505-w

**Published:** 2025-07-22

**Authors:** Roman Shendrik, Evgeny Radzhabov, Alexandra Myasnikova, Viktorija Pankratova, Anatolijs Šarakovskis, Alexander Nepomnyashchikh, Alexander Bogdanov, Veronika Gavrilenko, Ekaterina Kaneva, Dmitry Sofich, Tatiana Garmysheva, Vladimir Pankratov

**Affiliations:** 1 Vinogradov Institute of Geochemistry, SB RAS, Favorskii St. 1a, Irkutsk, 664033 Russia; 2 Institute of Solid State Physics, University of Latvia, 8 Kengaraga, Riga, LV-1063 Latvia

**Keywords:** Condensed-matter physics, Condensed-matter physics, Electronic structure, Optical materials

## Abstract

This study investigates the mechanisms underlying the ultrafast cross-luminescence observed in BaF_2_ crystals doped with LaF_3_. We identified an ultrafast luminescent component with a decay time of approximately 150 ps, which emerges under excitation energies exceeding 24 eV as a *novel radiative recombination process* between electrons in the 5p core band of Ba^2+^ and holes in the 5p core band of La^3+^. Ab initio calculations supported this hypothesis, showing that the energy levels of the core bands facilitate such transitions. The findings indicated that BaF_2_-LaF_3_ scintillators hold significant promise for the detection of ultrafast processes in high-energy physics and medical applications.

## Introduction

Cross-luminescence (or core-valence luminescence) was first discovered in BaF_2_ crystals in 1982^1^. This luminescence has unique properties such as fast decay (time constant of less than 1 ns) and no measured rise time. The mechanism of cross-luminescence was proposed in^2–4^, demonstrating that cross-luminescence occurs due to the excitation of the core 5p levels of the Ba^2+^ cations. Thus, cross-luminescence is characterized by an excitation threshold equal to the minimum energy of the transition of a 5p electron to the conduction band. Under such excitation, holes are formed in the Ba^2+^ core band, which can recombine with the electrons from the valence band. The oscillator strength of this radiative transition is high^5,6^, resulting in a very fast luminescence decay.

Later, cross-luminescence was observed in other wide-bandgap compounds containing Cs, K, Rb, and Ba^3,7–9^. The prerequisite for the occurrence of cross-luminescence is that the energy gap between the top of the core band and the bottom of the valence band should be smaller than the band gap (E_g_) of the compound. Due to their fast decay time, cross-luminescent materials are promising candidates for fast scintillators for high counting rate applications like muon detection in the mu2e project of the Fermi lab^10^ and other electromagnetic calorimeter modules^11^.

Recently, BaF_2_-based scintillators have shown promise for medical applications such as time-of-flight positron-emission tomography (TOF-PET) and computed tomography (TOF-CT)^12^. The critical challenge in TOF-PET is achieving a coincidence time resolution (CTR) of approximately 10 ps, which allows for an increase in spatial resolution to 1 mm in real time. Such an improvement in spatial resolution can drastically increase the signal-to-noise ratio in TOF-CT and significantly reduce the dose rate of gamma irradiation in patients by several orders of magnitude^13^.

For TOF applications, it is important that the cross-luminescence in BaF_2_ crystals has one of the highest light outputs compared with other cross-luminescent materials^3^, as well as compared with other fast emission types such as intra-band luminescence or Cherenkov radiation. One of the drawbacks of BaF_2_-based scintillators is the presence of a relatively slow luminescence peaking at about 290 nm, which is attributed to self-trapped excitons (STE). However, it can be suppressed using special band-pass filters and metamaterials^14–17^, although this method is ineffective due to light losses. An alternative suppression method of STE luminescence is co-doping of BaF_2_. It has been found that the best degradation of STE luminescence is observed if BaF_2_ crystals are doped by La^3+^^18–21^, Cd^2+^^22^, or Y^3+^^23^ ions.

Recently, an unpolished sample of BaF_2_-30 mol% LaF_3_ single crystal without any optical greasing demonstrated a CTR of 89 ± 3 ps. However, it is expected that the CTR of a polished sample with proper greasing can be reduced to 25 ps^24^. This is one of the best CTR parameters for existing TOF detectors. In addition, it was shown that La-doped BaF_2_ single crystals exhibit ultrafast luminescence with a decay time component of approximately 100 ps under 511 keV excitation. It is worth noting that the contribution to the ultrafast component in the decay kinetics increases in BaF_2_ crystals with higher concentrations of La^3+^ ions^24^. However, the origin as well as the mechanism of creation of this ultrafast luminescence in La-doped BaF_2_ have not been explained so far.

In order to identify the origin of the ultrafast decay component in the current study BaF_2_-LaF_3_ single crystals were studied by means of time-resolved emission end excitation spectroscopy. Luminescence excitation was carried out in the (VUV) and soft X-ray energy ranges with the idea of ​​determining the excitation threshold of the ultrafast component. First-principles calculations of the electronic structure were applied to deconvolute the obtained experimental data. Based on experimental and theoretical results, a new type of cross-luminescence responsible for ultrafast luminescence in BaF_2_-LaF_3_ single crystals has been proposed.

## Methodology

### Crystal growth

BaF_2_-LaF_3_ mixed crystals were synthesized via the Bridgman method. The raw material consisted of polycrystalline BaF_2_ and LaF_3_ combined in a precise stoichiometric ratio. A polycrystalline batch was prepared using the following procedure:

- **Purification of BaF**_**2**_: High-purity (99.999%) barium fluoride powder (Lanhit) underwent threefold recrystallization in a graphite crucible under a high vacuum of 10⁻⁴ Pa. Cadmium fluoride (CdF_2_) was added to the batch as a scavenging agent to remove impurities^25,26^.
- **Purification of LaF**_**3**_: Separately, high-purity (99.999%) lanthanum fluoride powder (LaF_3_) was recrystallized three times under identical conditions in another graphite crucible.

**Fig. 1:**
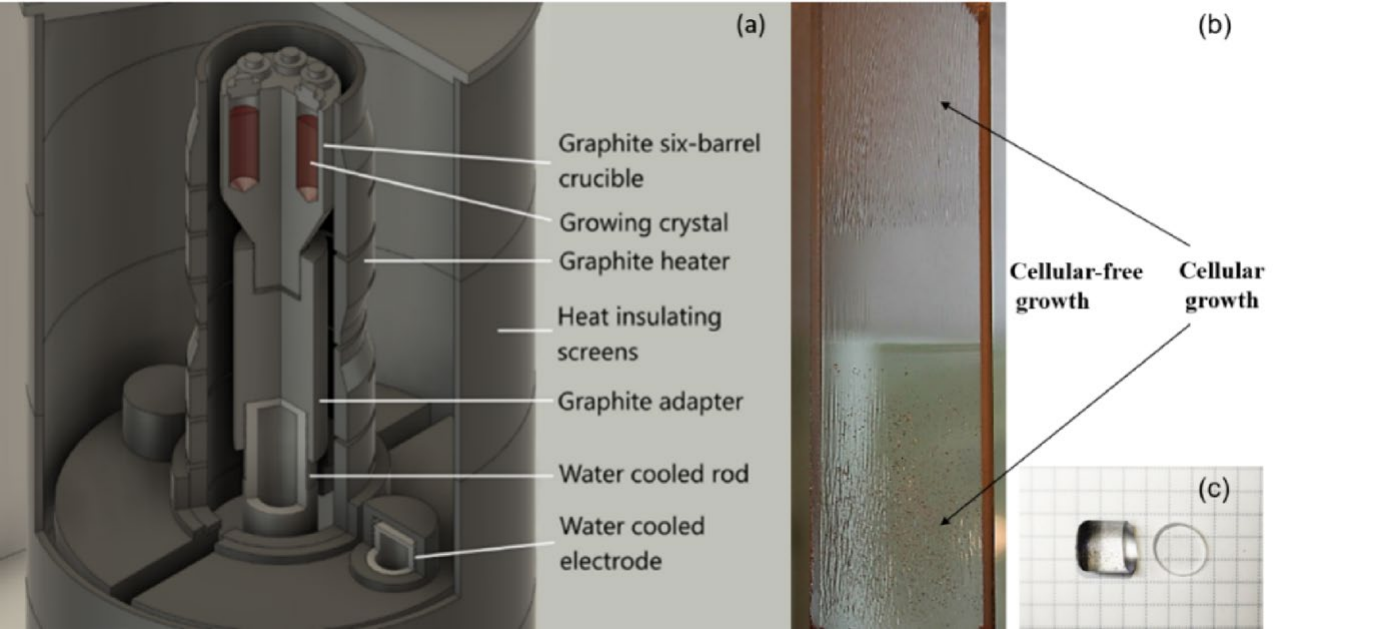
The crystal growth setup (**a**) and BaF_2_–30 mol% LaF_3_ crystal grown at different rates. The rate in the cellular growth region was 5 mm/h and the rate in the cellular free region was 1 mm/h (**b**). The growing crystal of BaF_2_ is given in (**c**).

After purification, the refined BaF_2_ batches were loaded into one- and multi-barrel crucible (Fig. 1a), with refined LaF_3_ added at the desired concentration. This approach enabled the growth of BaF_2_ crystals doped with variable lanthanum concentrations. Crystal growth was carried out under vacuum, with CdF_2_ added to the raw material as the scavenger agent. To determine optimal growth parameters, a single crystal was grown at a dynamically adjusted growth rate. The initial growth rate of 5 mm/h resulted in a cellular structure, which significantly degraded the optical homogeneity (Fig. 1b). Reducing the growth rate improved the crystal quality with an optimal rate of 1 mm/h. To minimize residual stress, the crystals underwent post-growth annealing. The lanthanum concentration in the final crystals was quantified using inductively coupled plasma mass spectrometry (ICP-MS) and atomic emission spectroscopy (ICP-AES). All crystals studied are colorless and transparent (Fig. 1c). The measured LaF_3_ levels matched the target doping concentrations, confirming precise compositional control. Undesirable oxygen impurities, which can easily contaminate fluorides, can be successfully controlled by luminescence techniques. If oxygen (O^2-^) replaces the fluorine anion, charge compensation occurs through the formation of an anion vacancy. In BaF_2_, the oxygen vacancy centers exhibit absorption bands at 5.2, 6.1, 7.2, and 8.4 eV. An intense, well-known photoluminescence peak at 2.38 eV is observed upon excitation within these absorption bands^27,28^. The absorption bands associated with oxygen impurities are located in the cross-luminescence region; hence, its intensity is reduced in oxygen-containing crystals due to the reabsorption of the cross-luminescence by the oxygen vacancy centers.

### Crystals’ characterization

X-ray diffraction (XRD) patterns were collected using a Bruker D8 ADVANCE (AXS, Berlin, Germany) diffractometer equipped with a scintillation detector. The measurements were performed in step-scan mode over a diffraction angle range (2θ) of 10 to 70° 2θ, employing a Cu Kα radiation source. The experiments were conducted at room temperature with a flat sample in the Bragg-Brentano geometry. The experimental conditions were as follows: 40 kV, 40 mA, exposure time − 2 s, step size − 0.02° 2θ. Data processing was performed using the DIFFRACplus software package (v. 2007, https://www.bruker.com/). Sample identification was performed using the Powder Diffraction File (PDF-2) database ICDD PDF-2, Release 2007, and indexing was carried out using the EVA software (v. 2007 (https://www.bruker.com/)^29^. The TOPAS 4.2 software package^30^ was used for the Rietveld profile fitting and unit cell parameter calculation. Rietveld refinements provided agreement factors of Rwp = 2.8–3.7%.

Raman spectra of crystals were obtained using a WITec alpha 300R confocal Raman spectroscopic system (WITec GmbH, Ulm, Germany) coupled with a 15 mW Nd: YAG laser (λ = 532 nm). The spectra were recorded in the range from 150 to 1200 cm^–1^ with a diffraction grating (1800 g mm^− 1^) and a spectral resolution of approximately 3 cm^− 1^.

### Spectroscopy

X-ray excited luminescence spectra were measured under excitation with a PD anode X-ray tube operated at 40 kV voltage and 2 mA current. The luminescence was registered with a VM4 evacuated monochromator and a photoelectron multiplier FEU-39 A with a quartz window.

Time-resolved spectra were measured at the photoluminescence endstation (*Finestlumi*)^31,32^ of the FinEstBeAMS beamline^33,34^ at the MAX IV synchrotron facility in Lund (Sweden). The measurements were performed in the single-bunch regime of the storage ring. Luminescence signals were registered using the Andor Shamrock (SR-303i) 0.3 m spectrometer coupled with a thermoelectrically cooled hybrid photodetector HPM-100–07 C (Becker & Hickl).

The intensity of each component in the decay was obtained through the deconvolution of the curves monitored at different wavelengths. The ideal decay time curve has a shape described by the following equation: $$\:{I}_{ideal}(\lambda\:,t)=\sum\:_{i=0}^{N}{A}_{i}\left(\lambda\:\right)\text{e}\text{x}\text{p}(-\raisebox{1ex}{$t$}\!\left/\:\!\raisebox{-1ex}{${\tau\:}_{i}\left(\lambda\:\right)$}\right.)$$. The real luminescence decay curve is $$\:{I}_{real}(\lambda\:,\:t)=IRF\left(t\right)⨂{I}_{ideal}(\lambda\:,\:t)$$, where IRF is the instrument response function. The intensity of each component at λ is $$\:{I}_{i}\left(\lambda\:\right)={{A}_{i}\left(\lambda\:\right)\tau\:}_{i}\left(\lambda\:\right)$$. An R^2^ of each fitting is not less than 0.99.

### Ab initio calculations

Ab initio calculations for crystals of mixed barium halides were carried out within the framework of density functional theory using the VASP software package^35^ on the “Akademik V.M. Matrosov” computing cluster^36^. For the calculations, a 2 × 2 × 2 supercell (96 atoms) was constructed in which one rare earth ion was substituted for one of the lattice cations. Atomic positions and crystal symmetry were obtained from the ICSD database^37^. Geometry optimization calculations were performed using the gradient approximation with the PBEsol exchange-correlation functional.

Currently, periodic calculations using the density functional method are performed within the framework of the gradient approximation using the exchange-correlation potential PBE or PBEsol. It is known that using the PBE density functional for calculations in semiconductors and insulators leads to delocalized electronic states and, consequently, underestimated bandgap energies^38^. The most accurate methods of the GW0 type^39^, which provide very good agreement with the experimental results for halide crystals, are also gaining popularity, however, these methods require substantial computational resources. Additionally, DFT calculations were conducted using hybrid functionals (PBE0, HSE), yielding good agreement with the experimental data in the previous works^40,41^.

## Results and discussion

### Characterizations of the crystals

**Fig. 2:**
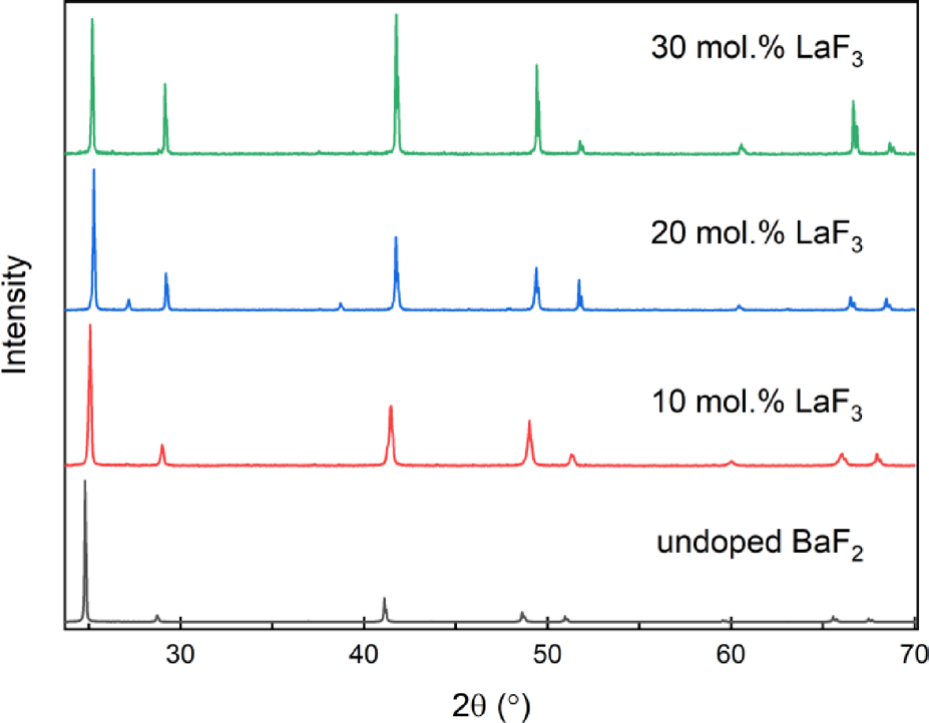
Powder diffraction patterns of undoped BaF_2_ and BaF_2_ – xLaF_3_ solid solutions (x = 0; 10; 20 and 30 mol%).

The unit cell parameters of the BaF_2_-LaF_3_ crystals were examined by XRD (Fig. 2). With increasing the LaF_3_ concentration, the unit cell parameter *a* decreased slightly without changes in the *Fm-3m* space group (Fig. 2). For BaF_2_–30 mol% LaF_3_, the values amount to *a* = 6.161(6) Å, V = 233.8(4) Å^3^ in comparison with *a* = 6.193(3) Å and V = 237.5(1) Å^3^ for the undoped BaF_2_.

**Fig. 3:**
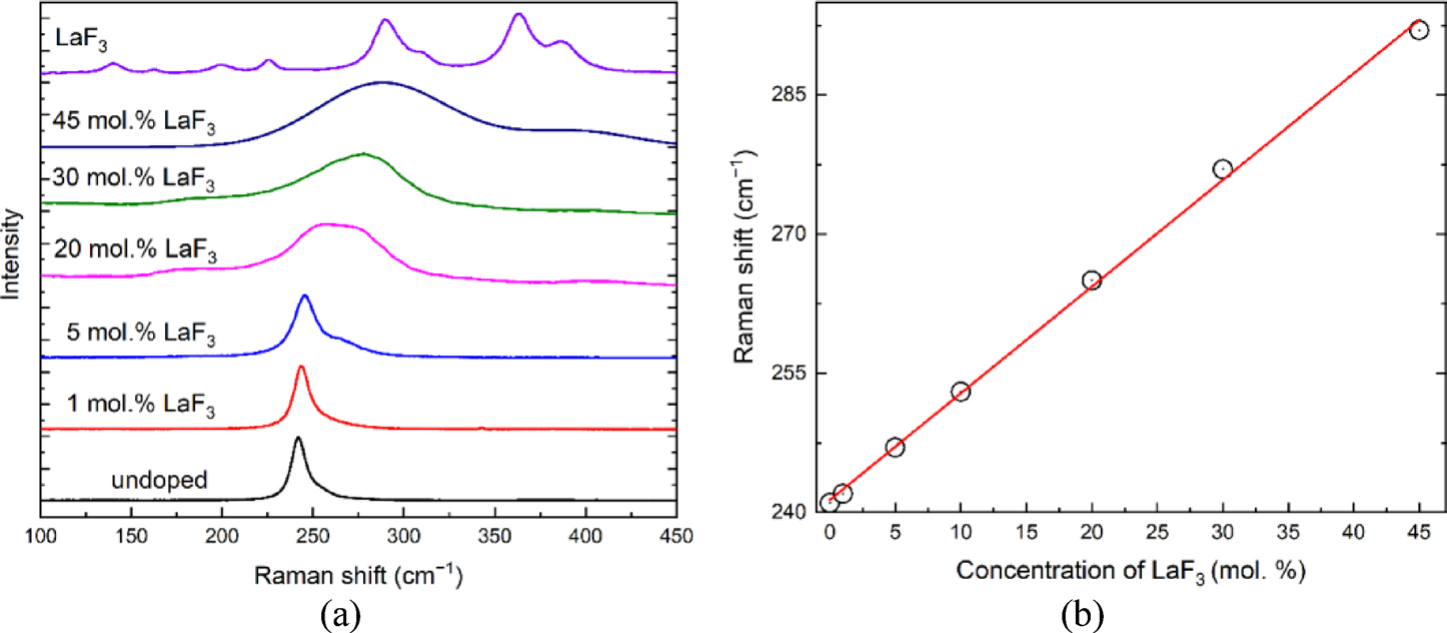
Raman spectra of BaF_2_ – xLaF_3_ solid solutions. For comparison, the Raman spectrum of the LaF_3_ single crystal (curve 5) (**a**), and the dependency with LaF_3_ mole fraction of the Raman peak position of the BaF_2_ – xLaF_3_ solid solutions (**b**) are given.

The Raman spectra (Fig. 3a) confirm a decrease in the unit cell parameter of the BaF_2_-LaF_3_ crystals. During the doping process, La^3+^ ions occupy the Ba^2+^ lattice sites, with charge compensation achieved through the incorporation of the interstitial fluorine ions. At higher La^3+^ concentrations, this leads to a slight disordering of the fluorine anion sublattice, and a consequent change in the unit cell parameters as calculated from the XRD results.

As pointed out in^42^, BaF_2_ − xLaF_3_ (x = 0–50 mol%) forms solid solution crystals with a fluorite structure and exhibits fluorine disordering when x > 10 mol%. The Raman spectra of crystals with varying x values are shown in Fig. 3a. For comparison, the LaF_3_ spectrum with a tysonite structure is also included. As the proportion of LaF_3_ in the BaF_2_-xLaF_3_ solid solution increases, the Raman frequency of the lattice vibrations rises. The full width at half maximum (FWHM) of the Raman bands of the BaF_2_-xLaF_3_ crystals, where x > 10 mol%, is twice that of the undoped BaF_2_ single crystal. This increase is attributed to fluorine disordering, as the fluorine anions require charge compensation for the La^3+^ ions substituting the Ba^2+^ cations. The band shift follows Vegard’s law, suggesting that lanthanum and barium are mixed homogeneously and randomly at the cation sites in the crystal with the fluorite structure (Fig. 3b).

### Ab initio calculations

**Fig. 4:**
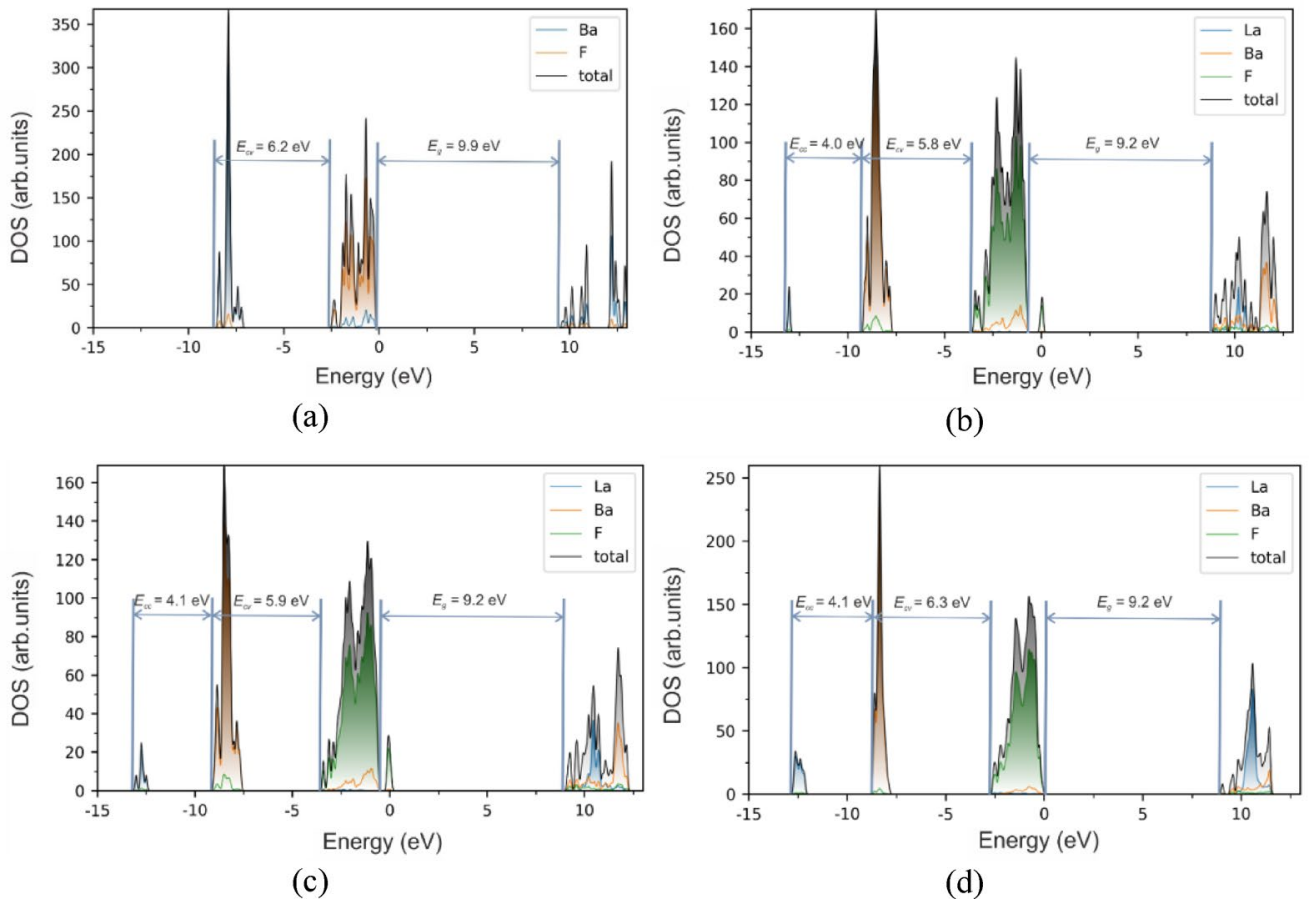
Density of States and Projected Density of States of undoped BaF_2_ (**a**); BaF_2_ – 10 mol% LaF_3_ (**b**); BaF_2_ – 20 mol% LaF_3_ (**c**) and BaF_2_ – 30 mol% LaF_3_ (**d**).

The calculation results are presented in Fig. 4. Initially, the parameters were calculated for a defect-free BaF_2_ crystal using PBE0 hybrid functional. The band gap of the crystal, estimated as the distance between the upper occupied and lower unoccupied states, was found to be 9.9 eV for undoped BaF_2_ and 9.2 eV for BaF_2_ doped with LaF_3_, which is about 1.09 times less than the experimental value. However, this method provides more accurate values than PBEsol, which underestimates the band gap by more than two times. The valence band is predominantly formed by the 2p states of the regular fluorine ions, whereas the upper core band is formed by the 5p states of the barium ions. An increase in LaF_3_ content leads to a decrease in the lattice constant. However, this change does not have a significant impact on the band gap value.

When a lanthanum atom is introduced into the lattice, 5p levels of the lanthanum ions are introduced below the Ba core band. The 2p levels of interstitial fluorine ions, which are charge compensators of La^3+^ are located in the forbidden band of the crystal (Fig. 4b – d). As the La^3+^ concentration increases, the 5p levels of La^3+^ expand into the subband. The energy difference between the bottom of the 5p La^3+^ band and the bottom of the conduction band is approximately 22 eV. The calculated distance between the bottom of the 5p Ba^2+^ band and the bottom of the conduction band is about 18 eV. These values are important for further interpretation of the excitation threshold of ultrafast luminescence.

Ab initio calculations showed that the 5p La^3+^ core states are located below the core band of Ba^2+^. The calculated energy gap between the bottom of the 5p Ba^2+^ core band and the 2p F^−^ valence band is about *E*_*cv*_ = 5.9–6.3 eV, whereas the distance between the bottom of the 5p Ba^2+^ core band and core 5p La^3+^ band is about *E*_*cc*_ = 4.1 eV (Fig. 4). Thus, the relationship *E*_*cc*_ *< E*_*cv*_ *< E*_*g*_, where *E*_*g*_ is the calculated band gap (9.2 eV), is satisfied, indicating that radiative core-to-core transitions between the core 5p levels of La^3+^ and Ba^2+^ could occur. The probability of these transitions increases with the widening of the La^3+^ subband in crystals doped with high concentrations of La^3+^. The position of the maximum of the ^5^p_3/2_ level of La^3+^ in the LaF_3_ crystal is approximately 1.5 eV lower^43^ than that of the ^5^p_3/2_ level of Ba^2+^ in the BaF_2_ crystal^44^, which is quite close to our calculations. In the^45^ ab initio calculations of band gap and core-valence gap give the larger values at about 20% than in the present research. However, the relationship between the gaps is the same to the given results. The differences in the absolute gap values obviously are due to different calculation techniques applied.

### Luminescence spectroscopy

**Fig. 5:**
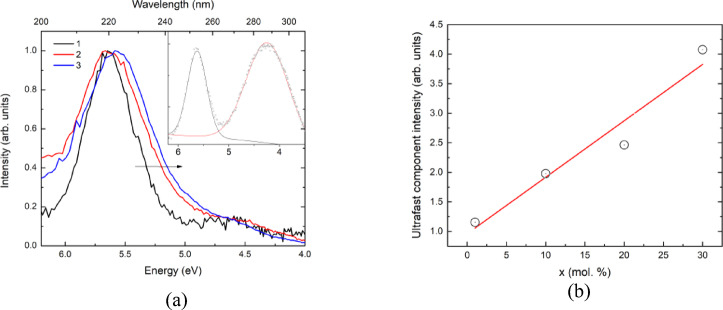
The figure (**a**) presents X-ray excited luminescence of the undoped BaF_2_ single crystal (1) and the doped – 30 mol% LaF_3_ (2) and 45 mol% LaF_3_ (3). The exciton band was subtracted from the curve (1) for clarity. Inset of figure (**a**) shows X-ray excited luminescence spectrum of undoped BaF_2_ without subtraction of STE band. The figure (**b**) demonstrates the concentration dependence of the ultrafast decay time component intensity (circles) under 511 KeV excitation^24^ in the BaF_2_ – xLaF_3_ system (x = 1; 10; 20; and 30 mol%).

The luminescence spectra of the undoped BaF_2_ and the BaF_2_ doped with 30 and 45 mol% LaF_3_ under X-ray excitation are shown in Fig. 5a. In the undoped BaF_2_, two luminescence bands are observed in the 3–6 eV range, with maxima at 4.3 eV (288 nm) and 5.6 eV (220 nm) (Fig. 5a, inset). The 4.3 eV band corresponds to self-trapped excitons (STE) and exhibits a decay time of approximately 600 ns^46^. The second band at 5.6 eV is attributed to regular cross-luminescence, characterized by a decay time shorter than 1 ns^47^.

Doping with LaF_3_ suppresses the STE luminescence (Fig. 5a, curves 2, 3) and induces a redshift of the cross-luminescence band with increasing LaF_3_ concentration. For clarity, the STE band was subtracted from the undoped BaF_2_ spectrum in Fig. 5a, revealing the redshift and broadening explicitly. The concentration-dependent redshift of cross-luminescence is quantified in Fig. 5b. The intensity of the ultrafast component increases with the concentration of LaF_3_. However, in samples containing more than 30 mol% LaF_3_, the total cross-luminescence intensity decreases significantly. Therefore, the optimal concentration is approximately 20 mol%.

**Fig. 6:**
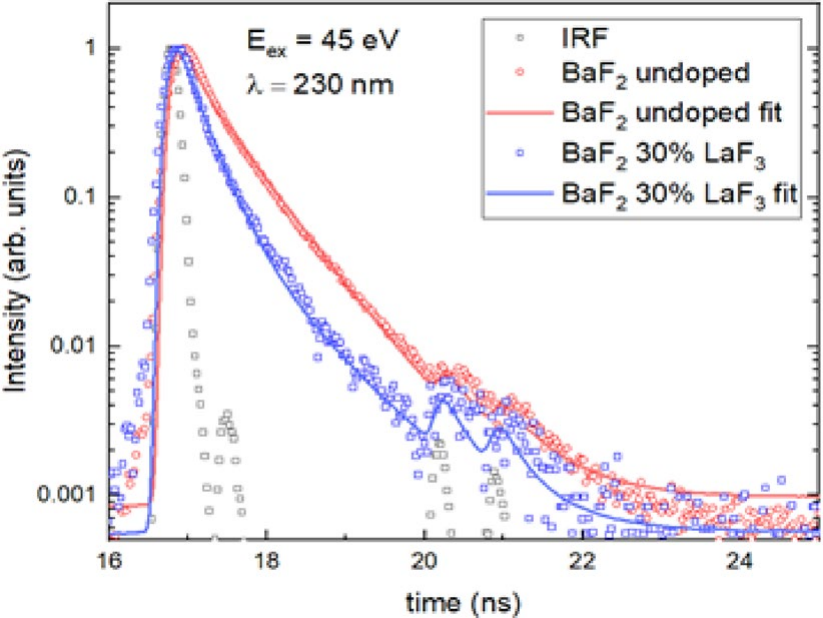
Decay time curves of undoped BaF_2_ and BaF_2_-30 mol% LaF_3_ under 45 eV excitation at 230 nm.

Figure 6 compares the decay time curves of nominally pure BaF_2_ and BaF_2_ doped with 30 mol% LaF_3_. In undoped BaF_2_ single crystals, the fast luminescence component (decay time constant ≈ 650 ps) is associated with the intrinsic (regular) cross-luminescence, while the long-lived component (600 ns) corresponds to STE emission (Table 1).

**Table 1:** Decay time curve analysis of undoped BaF_2_ and BaF_2_-30 mol% LaF_3_ under 45 ev excitation.

	λ, nm	τ_1_, ns	I_1_, %^*)^	τ_2_, ns	I_2_, %	τ_3_, ns	I_3_, %
BaF_2_	230			0.65 ± 0.03	89 ± 0.6	600 ± 2	11 ± 0.5
BaF_2_ – 30 mol% LaF_3_	230	0.15 ± 0.03	0.7 ± 0.3	0.65 ± 0.03	99.3 ± 0.6		
	260	0.15 ± 0.02	2 ± 0.4	0.65 ± 0.03	88 ± 0.7	10 ± 1	10 ± 0.5

^*)^ I_i_ – an integral intensity of each *i* component. $\:{I}_{i}\left(\lambda\:\right)={{A}_{i}\left(\lambda\:\right)\tau\:}_{i}\left(\lambda\:\right)$. The intensities $\:{I}_{i}\left(\lambda\:\right)$ were corrected on quantum efficiency of the detector.

An ultrafast luminescence component emerges in the BaF_2_-LaF_3_ single crystals under excitation energies exceeding 24 eV. The UV luminescence at 220–240 nm (cross-luminescence) exhibits two distinct decay components. One of them is a decay component with a time constant of approximately 650 ps, consistent with regular cross-luminescence, whereas another one is an ultrafast component exhibiting a significantly shorter decay time constant of ~ 150 ps (see Table I for details). This ultrafast component observed in BaF_2_–30 mol% LaF_3_ contributes ~ 1% of the total UV luminescence intensity.

The contribution of the ultrafast component to the cross-luminescence increases with the excitation energy. Under 511 keV excitation, it accounts for up to 30% of the total cross-luminescence intensity^24^. Using data from^24^, the concentration-dependent intensity of the ultrafast component under 511 keV excitation was plotted (Fig. 5b, circles). A clear correlation is observed between the redshift of the observed cross-luminescence in Fig. 5a and the increase in the relative intensity of the ultrafast component under 511 keV excitation.

**Fig. 7:**
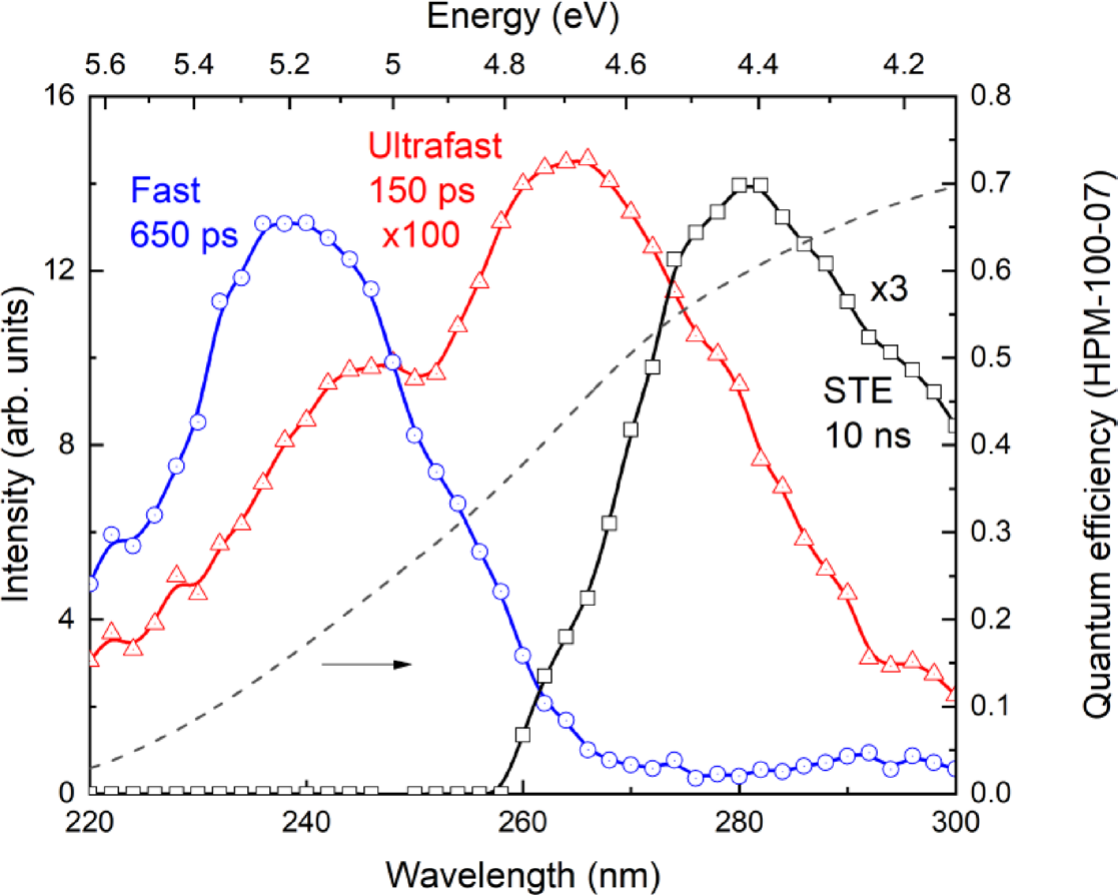
Time-resolved spectra of ultrafast 150 ps (red curve) and fast 650 ps (blue curve) components of cross-luminescence under 45 eV excitation in BaF_2_-30 mol% LaF_3_. The black curve shows the STE-related luminescence. The dashed curve represents the quantum efficiency of the HPM-100-07 photodetector. The time-resolved spectra were not corrected with respect the quantum efficiency of the detector.

The time-resolved cross-luminescence emission spectra of the 150 ps and 650 ps components are shown in Fig. 7. The 150 ps ultrafast luminescence time-resolved spectrum contains two bands peaking at approximately 245 nm and 270 nm. Both ultrafast 150 ps luminescence bands are redshifted in respect of regular fast cross-luminescence band peaking at 235 nm. The STE luminescence band is located around 280 nm, with a decay time constant of around 10 ns due to concentration quenching of the STE luminescence in the BaF_2_-30 mol% LaF_3_ crystal.

**Fig. 8:**
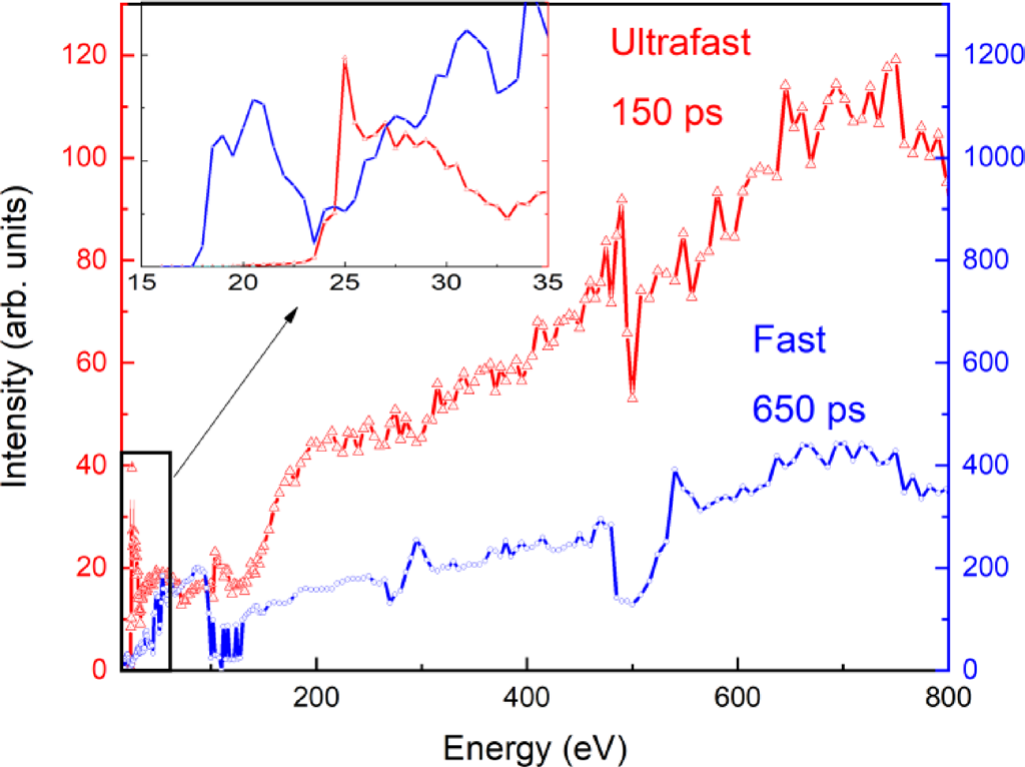
Excitation spectra of the fast (650 ps) and ultrafast (150 ps) components of cross-luminescence monitored at 230 nm.

The excitation spectra of the 150 ps and 650 ps luminescence components are presented in Fig. 8. The 650 ps component is excited at energies higher than the edge of 18 eV, which corresponds to the energy transition from the core 5p level of Ba to the 5d states that form the conduction band. The excitation edge for the 150 ps component is shifted to a higher energy region, located at approximately 23.5 eV.

The behavior of the excitation spectra for the 150 ps and 650 ps components differs. In the range of 50–800 eV, the intensity of 650 ps component is poorly dependent on excitation energy. However, the intensity of 150 ps component demonstrates a significantly stronger monotonous increase. If we extrapolate such an increase to the keV energy range, the contribution of the ultrafast component will be quite large. This suggestion is supported by the results reported in^24^, where the contribution of the ultrafast component increases to 20–30% of the total intensity under 511 keV excitation.

The various components involved in cross-luminescence decay were identified in undoped BaF_2_ materials. The quenching mechanism was attributed to surface quenching and energy transfer to excitations generated by the same photon. Surface quenching plays a significant role at energies below 30 eV and is influenced by the angle of the incident beam. The estimated penetration depth of excitation photons in BaF_2_ is approximately 44 Å for 30 eV excitation^48^. At higher energies, energy transfer from the core hole to other electronic excitations may become the dominant process. The absolute value of the quenching component is energy-dependent, and the decay time constant of the quenched component decreases as energy increases. No correlation was found between cross-luminescence quenching and the presence of dopants^48^. Furthermore, the behavior of the quenched component differs from the ultrafast component observed in BaF_2_-LaF_3_ crystals in this study, indicating that it cannot be attributed to quenching processes.

It was shown in Fig. 4 ab initio calculations predicted the formation of a core band composed of the 5p states of La^3+^ ions. On the other hand, the observed redshift in the cross-luminescence is strongly dependent on lanthanum concentration (Fig. 5b). Thus, we suggest that 5p states of La^3+^ are connected with the red-shifted cross-luminescence and, most likely, radiative recombination between the core bands of Ba^2+^ and La^3+^ ions becomes feasible. In what follows we will refer to such luminescence as *core-to-core luminescence* in order to distinguish it from regular cross-luminescence or core-valence luminescence. It is worth noting that the energy gap between the core bands of Ba^2+^ and La^3+^ is smaller than that between the Ba^2+^ core and valence bands. As a result, the core-to-core luminescence exhibits a redshift compared with regular cross-luminescence. Considering that the ultrafast 150 ps luminescence component is also red-shifted relative to the regular cross-luminescence band (Fig. 7), it would be quite natural to associate it with core-to-core luminescence.

**Fig. 9:**
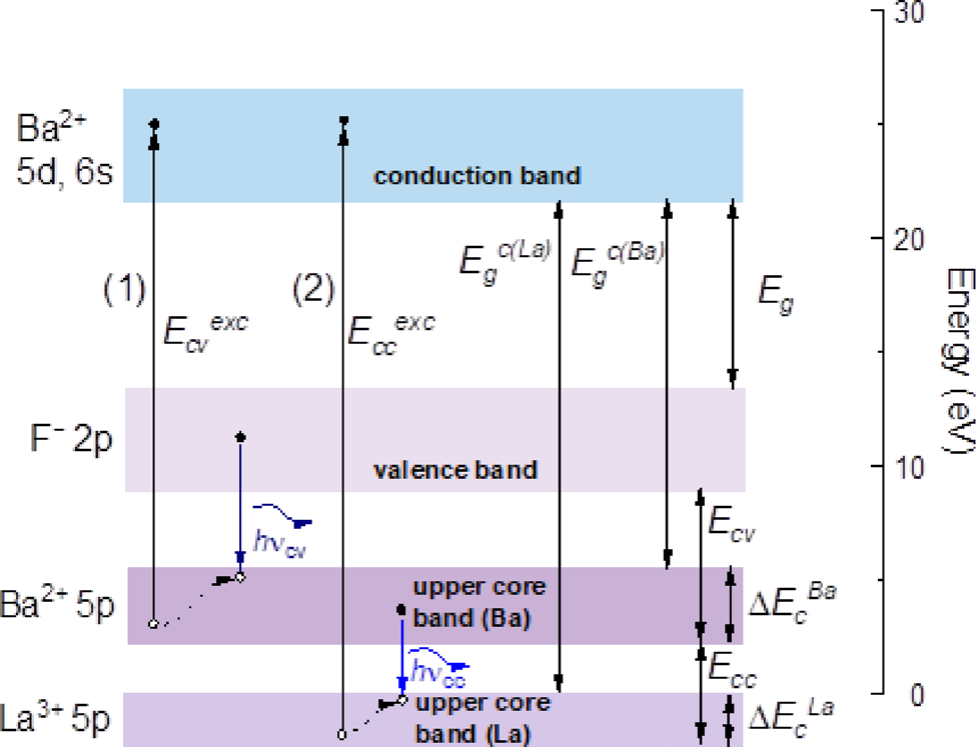
The simplified mechanism of core-valence (*h*ν_cv_) (τ = 650 ps) and core-to-core (*h*ν_cc_) (τ = 150 ps) luminescence.

The simplified model of this process is shown in Fig. 9. Under the excitation of the core 5p levels of Ba^2+^ with energies higher than *E*_*cv*_^*exc*^ >17 eV, regular 650 ps cross-luminescence occurs due to the recombination of electrons from the valence band and holes in the core Ba^2+^ band (*hυ*_cv_). In BaF_2_-LaF_3_ crystals, the core band of La³⁺ lies deeper in energy than that of the Ba^2+^ ions, implying that the excitation energy required for the La^3+^ core should be higher. The threshold energy for the excitation of the 150 ps luminescence component is *E*_*cc*_^*exc*^>24 eV (Fig. 8), which is close to the excitation threshold of the core 5p-La^3+^ band estimated from the calculations (*E*_*g*_^*c(La)*^ = 21 eV) and higher than the *E*_*g*_^*c(Ba)*^ = 17 eV excitation threshold of the regular 650 ps luminescence. This result is completely consistent with our assumption that the ultrafast 150 ps luminescence is associated with the radiative recombination of electrons from the 5p Ba^2+^ core band with holes in the 5p La^3+^ core band (*h*ν_cc_). The model depicted in Fig. 9 clearly demonstrates that the energy gap between the 5p core bands of Ba^2+^ and La^3+^ *(E*_*cc*_) is less than the gap between the 5p Ba^2+^ core band and the 2p F^−^ valence band (*E*_*cv*_), explaining the redshift of the core-to-core luminescence discussed above.

The width of the La^3+^ 5p core band (Δ*E*_*c*_^*La*^=1.88 eV) is less than the width of the 5p Ba^2+^ core band (Δ*E*_*c*_^*Ba*^=2.56 eV), according to our calculations. In this case, according to^43,47^, the hole relaxation process in 5p La^3+^ core band occurs faster than in 5p Ba^2+^ core band. This could explain the shorter decay time of core-to-core luminescence compared with the regular cross-luminescence.

The double peaks structure in the fast 150 ps core-to-core luminescence spectrum shown in Fig. 7 could arise from the spin-orbital coupling of 5p Ba^2+^ upper core band into two core levels ^5^p_1/2_ and ^5^p_3/2_^44^. Another explanation could be related to the transitions between the 5p-orbitals of La^3+^ and the 2p orbitals of the nearest fluorine ions^6^.

## Conclusion

Ultrafast luminescence with a decay time constant of 150 ps in BaF_2_-LaF_3_ crystals has been observed for the first time under VUV excitations. It exhibits a doble peak emission structure at 245 nm and 270 nm revealing a significant redshift compared to the regular 650 ps cross-luminescence (peaking at 230 nm). It was demonstrated that the ultrafast 150 ps luminescence has an excitation threshold of 24 eV. The excitation spectrum in VUV and soft X-ray range demonstrate a confident growth of the ultrafast luminescence with a tendency to reach a strong intensity under keV excitations. Based on these experimental data as well as on ab initio calculations we propose that this ultrafast luminescence is explained by the core-to-core recombination of an electron from the 5p Ba^2+^ band and a hole in the 5p La^3+^ band. It is also suggested that the ultrafast decay time of core-to-core luminescence is due to fast hole relaxation in the La^3+^ core band.

## Electronic supplementary material

Below is the link to the electronic supplementary material.

Supplementary Material 1

## Data Availability

The authors declare that the data supporting the findings of this study are available within the paper, its supplementary information files.
